# Supplementary material for: Modeling glioblastoma heterogeneity as a dynamic network of cell states
Source: Mol Syst Biol. 2021 Sep 16;17(9):e10105. doi: 10.15252/msb.202010105 (PMC8444284; doi:10.15252/msb.202010105)
Supplement: Supplementary file 6 — Source Data for Figure 5 [file MSB-17-e10105-s004.zip › Figure5A_sourcedata/GSEA_3071/nosigdb_stateIII.GseaPreranked.1622795155562/FELDMAN_2020_G1.html]

Details for gene set FELDMAN\_2020\_G1[GSEA]

|  || Dataset | state33071 |
| Phenotype | NoPhenotypeAvailable |
| Upregulated in class | na\_neg |
| GeneSet | FELDMAN\_2020\_G1 |
| Enrichment Score (ES) | -0.5007236 |
| Normalized Enrichment Score (NES) | -2.8083627 |
| Nominal p-value | 0.0 |
| FDR q-value | 0.0 |
| FWER p-Value | 0.0 |
Table: GSEA Results Summary

  

Fig 1: Enrichment plot: FELDMAN\_2020\_G1      
 Profile of the Running ES Score & Positions of GeneSet Members on the Rank Ordered List

  

| PROBE | GENE SYMBOL | GENE\_TITLE | RANK IN GENE LIST | RANK METRIC SCORE | RUNNING ES | CORE ENRICHMENT || 1 | CYCS |  |  | 59 | 0.453 | -0.0011 | No |
| 2 | CALM2 |  |  | 120 | 0.396 | -0.0042 | No |
| 3 | SNRPB |  |  | 122 | 0.395 | 0.0078 | No |
| 4 | C12orf75 |  |  | 203 | 0.337 | -0.0023 | No |
| 5 | RANBP1 |  |  | 221 | 0.328 | 0.0035 | No |
| 6 | PTMA |  |  | 222 | 0.327 | 0.0137 | No |
| 7 | PTGES3 |  |  | 252 | 0.310 | 0.0159 | No |
| 8 | STMN1 |  |  | 298 | 0.291 | 0.0134 | No |
| 9 | HMGB1 |  |  | 318 | 0.286 | 0.0174 | No |
| 10 | SNRPD1 |  |  | 376 | 0.268 | 0.0111 | No |
| 11 | H2AFZ |  |  | 386 | 0.265 | 0.0170 | No |
| 12 | RAN |  |  | 388 | 0.264 | 0.0250 | No |
| 13 | NDC80 |  |  | 636 | -0.261 | -0.0307 | No |
| 14 | DHFR |  |  | 956 | -0.277 | -0.1044 | No |
| 15 | CDCA3 |  |  | 1029 | -0.281 | -0.1142 | No |
| 16 | RNASEH2A |  |  | 1103 | -0.285 | -0.1242 | No |
| 17 | PCNA |  |  | 1116 | -0.286 | -0.1184 | No |
| 18 | NUSAP1 |  |  | 1262 | -0.293 | -0.1467 | No |
| 19 | GGH |  |  | 1439 | -0.302 | -0.1827 | No |
| 20 | KIF4A |  |  | 1504 | -0.306 | -0.1897 | No |
| 21 | DEPDC1 |  |  | 1564 | -0.310 | -0.1952 | No |
| 22 | NUF2 |  |  | 1755 | -0.322 | -0.2343 | No |
| 23 | PLK1 |  |  | 1828 | -0.326 | -0.2427 | No |
| 24 | UBE2C |  |  | 1942 | -0.332 | -0.2615 | No |
| 25 | SMC2 |  |  | 1963 | -0.333 | -0.2563 | No |
| 26 | RRM2 |  |  | 2147 | -0.345 | -0.2928 | No |
| 27 | TPX2 |  |  | 2289 | -0.356 | -0.3181 | No |
| 28 | DLG1 |  |  | 2317 | -0.359 | -0.3139 | No |
| 29 | DLGAP5 |  |  | 2421 | -0.367 | -0.3290 | No |
| 30 | PRCP |  |  | 2489 | -0.372 | -0.3347 | No |
| 31 | CCNA2 |  |  | 2635 | -0.385 | -0.3601 | No |
| 32 | KIF20B |  |  | 2689 | -0.390 | -0.3616 | No |
| 33 | SMC4 |  |  | 2872 | -0.405 | -0.3960 | No |
| 34 | ATAD2 |  |  | 3115 | -0.433 | -0.4450 | No |
| 35 | AHNAK2 |  |  | 3190 | -0.442 | -0.4503 | No |
| 36 | CDCA2 |  |  | 3212 | -0.444 | -0.4419 | No |
| 37 | TPST2 |  |  | 3437 | -0.489 | -0.4845 | Yes |
| 38 | GTSE1 |  |  | 3468 | -0.498 | -0.4767 | Yes |
| 39 | NCAPG |  |  | 3513 | -0.512 | -0.4720 | Yes |
| 40 | ANLN |  |  | 3625 | -0.562 | -0.4832 | Yes |
| 41 | TOP2A |  |  | 3671 | -0.594 | -0.4762 | Yes |
| 42 | CENPE |  |  | 3711 | -0.625 | -0.4668 | Yes |
| 43 | HIST1H1B |  |  | 3749 | -0.669 | -0.4555 | Yes |
| 44 | ARL6IP1 |  |  | 3759 | -0.685 | -0.4364 | Yes |
| 45 | SCG2 |  |  | 3786 | -0.718 | -0.4207 | Yes |
| 46 | MKI67 |  |  | 3788 | -0.721 | -0.3984 | Yes |
| 47 | MIAT |  |  | 3833 | -0.794 | -0.3850 | Yes |
| 48 | CENPF |  |  | 3842 | -0.820 | -0.3614 | Yes |
| 49 | GJB2 |  |  | 3845 | -0.826 | -0.3361 | Yes |
| 50 | S100B |  |  | 3853 | -0.845 | -0.3116 | Yes |
| 51 | SCRG1 |  |  | 3869 | -0.904 | -0.2872 | Yes |
| 52 | PTN |  |  | 3884 | -1.022 | -0.2589 | Yes |
| 53 | CYR61 |  |  | 3887 | -1.037 | -0.2270 | Yes |
| 54 | IGFBP5 |  |  | 3895 | -1.082 | -0.1951 | Yes |
| 55 | ASPM |  |  | 3899 | -1.166 | -0.1594 | Yes |
| 56 | CTGF |  |  | 3901 | -1.185 | -0.1226 | Yes |
| 57 | IGFBP3 |  |  | 3903 | -1.199 | -0.0855 | Yes |
| 58 | HIST1H4C |  |  | 3914 | -1.352 | -0.0458 | Yes |
| 59 | MALAT1 |  |  | 3924 | -1.583 | 0.0013 | Yes |
Table: GSEA details [plain text format]

  

Fig 2: FELDMAN\_2020\_G1: Random ES distribution      
 Gene set null distribution of ES for **FELDMAN\_2020\_G1**
